# Association of Blood Type With Postsurgical Mucosal Bleeding in Pediatric Patients Undergoing Tonsillectomy With or Without Adenoidectomy

**DOI:** 10.1001/jamanetworkopen.2020.1804

**Published:** 2020-03-31

**Authors:** Natasha M. Archer, Peter W. Forbes, Jenna Dargie, Juliana Manganella, Greg R. Licameli, Margaret A. Kenna, Carlo Brugnara

**Affiliations:** 1 Pediatric Hematology, Oncology Dana-Farber, Children’s Hospital Blood Disorders and Cancer Center, Boston, Massachusetts; 2 Clinical Research Center, Boston Children's Hospital, Boston, Massachusetts; 3 Department of Otolaryngology and Communication Enhancement, Boston Children’s Hospital, Boston, Massachusetts; 4 Laboratory Medicine, Boston Children's Hospital, Boston, Massachusetts

## Abstract

**Importance:**

Blood type (BT) O has been identified as a risk factor for bleeding complications, while non-O BTs may increase risk for thromboembolic events. Limited data are available in children undergoing tonsillectomy with or without adenoidectomy.

**Objective:**

To determine whether BT O is associated with hemorrhage after tonsillectomy with or without adenoidectomy.

**Design, Setting, and Participants:**

Retrospective cohort study of patients younger than 22 years who underwent tonsillectomy with or without adenoidectomy at a single institution between January 1, 2008, and August 7, 2017. Statistical analysis was performed from November 2017 to January 2019.

**Main Outcomes and Measures:**

Prevalence of hemorrhage following surgery was defined as any bleeding requiring cauterization up to 1 month after the procedure. Data on sex, age, von Willebrand disease (VWD) status, BT, white blood cell counts, and platelet counts closest to date of surgery were collected from an electronic medical record system, and the association of these factors with hemorrhage following surgery was investigated.

**Results:**

A total of 14 951 pediatric patients (median [range] age, 5.6 [0.8-21.9] years; 6956 [46.5%] female) underwent tonsillectomy with or without adenoidectomy. Prevalence of hemorrhage following the procedure was 3.9% (578 patients) for the full cohort and 2.8% (362 of 13 065) for patients with no BT identified or preprocedure VWD panel results at baseline. Children who had a BT identified and/or a VWD panel before surgery had higher bleeding rates (BT only, 14.9% [172 of 1156]; preprocedure VWD panel only, 4.6% [28 of 607]; and BT and preprocedure VWD panel, 13.0% [16 of 123]), all of which were significantly different from the baseline bleeding rate (*P* < .001). While the bleeding rates in children with BT O were not statistically different from those with non-O BT (14.8% and 14.6%, respectively; *P* > .99), mean von Willebrand factor values were statistically different (mean [SD] von Willebrand factor antigen level in O group, 86.9 [42.4] IU/dL in the O group vs 118.0 [53.8] IU/dL in the non-O group; *P *= .002; and mean [SD] von Willebrand factor ristocetin-cofactor in the O group, 72.2 [44.3] IU/dL vs 112.6 [68.0] IU/dL in the non-O group; *P* = .001). In addition, children older than 12 years had increased bleeding rates in the full cohort (8.3% vs 3.2%), in the testing-naive cohort (6.5% vs 2.3%), and in those with a preprocedure VWD panel only (13.5% vs 3.1%) compared with children aged 12 years or younger.

**Conclusions and Relevance:**

Type O blood was not a risk factor associated with hemorrhage after tonsillectomy with or without adenoidectomy despite lower baseline von Willebrand factor antigen and von Willebrand factor ristocetin-cofactor values in children with BT O vs those with non-O BT in our study cohort. No association was found between VWD status and bleeding, and there was no difference in VWD panel values in those who experienced hemorrhage vs those who did not within BT groups. Further studies elucidating the utility of von Willebrand factor values for children undergoing tonsillectomy with or without adenoidectomy are needed.

## Key Points

**Question:**

Is blood type O associated with a higher prevalence hemorrhage in children after tonsillectomy with or without adenoidectomy?

**Findings:**

In this cohort study of nearly 15 000 children who underwent tonsillectomy with or without adenoidectomy, the hemorrhage rate following surgery was 3.9%. In the cohort of children for whom a blood type was known, those with blood type O experienced bleeding as frequently as those with non-O types, despite having significantly lower baseline von Willebrand factor values.

**Meaning:**

The findings of this study indicate that bleeding rates after tonsillectomy with or without adenoidectomy are similar in children with blood type O and those with non-O types.

## Teaser

This cohort study examines whether blood type O is associated with hemorrhage after tonsillectomy with or without adenoidectomy in children.

## Introduction

In the United States, tonsillectomy is one of the most common surgical procedures in childhood.^1^ A review of the Nationwide Inpatient Sample between 2001 and 2010 found a 6.4% overall complication rate in US children and adolescents after tonsillectomy, with hemorrhage being a common complication (1.2%).^2^ Several potential risk factors have been identified, including increasing age,^3^ hot knife techniques,^4^ and tonsillitis as indication for tonsillectomy with or without adenoidectomy.^3,5^

In 1998, our institution established a preoperative bleeding questionnaire for all individuals scheduled for tonsillectomy with or without adenoidectomy. If a personal or family history of bleeding is identified, and if there are abnormal values in the prothrombin time, partial thromboplastin time, or low platelet count on the complete blood count, several preoperative coagulation screens are performed. If results are abnormal, children are referred to a pediatric hematologist.^6^

While von Willebrand disease (VWD) is the most common inherited blood disorder, with an estimated prevalence of approximately 1%, only 1 in 10 000 individuals have symptoms.^7,8^ Individuals with the disease have either decreased or abnormal von Willebrand factor (VWF), an essential primary hemostasis protein. Those who are symptomatic demonstrate mucocutaneous bleeding in the form of easy bruising, epistaxis, hematuria, bloody stool, menorrhagia, and mucocutaneous bleeding after trauma, including surgery. As part of the evaluation of a child with a suspected bleeding disorder, clinicians often request a VWD panel, which includes VWF antigen (VWF:Ag), VWF ristocetin-cofactor (VWF:RCo), and coagulation factor VIII value. Our group has shown that determination of blood type (BT) during the initial assessment of bleeding disorders decreased VWD panel testing in a cohort of children evaluated for VWD at our hospital.^9^

Blood group typing is not part of the preoperative testing for children undergoing tonsillectomy. There have been numerous reports of increased risk of bleeding in individuals with BT O,^10,11,12^ whereas non-O BT has been associated with a higher risk of thromboembolic events.^13^ While patients with BT O have lower values of VWF, it has not been demonstrated that these lower values are responsible for an increased bleeding risk. Paradoxically, there have also been reports that show no increased risk associated with BT O.^14,15,16,17^ We therefore questioned whether a BT assessment could help stratify children at risk for bleeding after tonsillectomy.

## Methods

We conducted a retrospective cohort study of all pediatric patients (aged <22 years) who underwent tonsillectomy with or without adenoidectomy as well as recauterization for bleeding from January 1, 2008, to August 7, 2017, at Boston Children’s Hospital in Boston, Massachusetts. Approval of the study protocol (including waiver of informed consent owing to minimal infraction of patient autonomy compared with benefit of knowledge gained) was obtained from the Boston Children’s Hospital institutional review board prior to data collection. Reporting follows the Strengthening the Reporting of Observational Studies in Epidemiology (STROBE) reporting guideline. In addition to age, sex, and occurrence of hemorrhage, preprocedure VWD panel values, BT, total white blood cell count, and platelet count closest to date of surgery were collected from our electronic medical record system. Two patients with hemophilia were excluded. Five patients with more than 1 BT in our system (secondary to bone marrow transplant) were also excluded. The second of 2 panels done on the same day (total of 32 panels) was excluded as it was part of a desmopressin challenge and often had results greater than baseline values.

The primary study outcome was incidence of hemorrhage following tonsillectomy with or without adenoidectomy, which was defined by a return for cautery to Boston Children’s Hospital within 1 month of initial operation. Additional variables collected included age; sex; BT; date of surgery; WBC and platelet count and dates; preprocedure VWF:Ag, VWF:RCo, and coagulation factor VIII values; and test date of VWD panel. These were used to assess the secondary outcome of risk factors associated with hemorrhage.

Patients were placed into 3 final VWD diagnosis categories (normal, low VWF, and disease) based on the results of preprocedure baseline panel testing extracted from the electronic medical record. To be classified as normal, all VWF:Ag and VWF:RCo values had to fall within the normal range (≥50 IU/dL), while low VWF (≥30 IU/dL and <50 IU/dL) or disease (<30 IU/dL) needed at least 1 value within their respective ranges to fall within the category. If a patient had panels that fell within the low VWF and disease value ranges, they were categorized as having disease.

Patients were further categorized into 4 main cohorts: (1) BT unknown and no preprocedure VWD panel, (2) BT known and no preprocedure VWD panel, (3) BT unknown and preprocedure VWD panel performed, and (4) BT known and preprocedure VWD panel performed.

Coagulation factor VIII, VWF:RCo, and VWF:Ag were measured on Siemens BCX and BCS-XP analyzers using Siemens Healthcare Diagnostics reagents (Innovance VWF Ac Kit and VWF Ag kit).

### Statistical Analysis

Data were extracted from the electronic medical record. Statistical analysis, performed November 2017 to January 2019, used SAS version 9.3 (SAS Institute Inc). The Fisher exact test was used to compare bleeding rates between groups. When continuous variables were compared between groups, the Wilcoxon test was used. A logistic regression was used to test independent associations of age, sex, VWD status (disease, low, normal, and unknown status) and BT (BT O, non-O BT, and unknown BT) with hemorrhage following tonsillectomy. Age was collapsed to 2 developmental levels (≤12 years and >12 years). The 1 patient with missing sex was excluded from the logistic regression. Two-sided *P* < .05 was considered statistically significant.

## Results

Surgeons at Boston Children’s Hospital performed a total of 14 951 tonsillectomies with or without adenoidectomy on children with a median (range) age of 5.6 (0.8-21.9) years. In all, 6956 patients (46.5%) were female (Table 1). A total of 578 patients (3.9%) had hemorrhage after surgery. Children older than 12 years experienced bleeding more often than younger ones (155 of 1860 [8.3%] vs 423 of 13 091 [3.2%]; *P* < .001) (Table 2).

**Table 1.** Characteristics of Study Cohort by Condition

Characteristic	No. (%)					*P* value
	All patients (N = 14 951)	No BT and no VWD panel (n = 13 065)	BT and no VWD panel (n = 1156)	No BT and VWD panel (n = 607)	BT and VWD panel (n = 123)	
Age at tonsillectomy, median (range), y	5.6 (0.8-21.9)	5.6 (0.8-21.9)	6.2 (1.0-21.9)	5.9 (0.9-21.8)	7.3 (1.1-21.4)	<.001
Female	6956 (46.5)	6070 (46.4)	554 (47.9)	278 (45.8)	54 (43.9)	.71
Patients with known BT (n = 1279)						
Type O	642 (50.2)	NA	555 (48.0)	NA	82 (66.7)	<.001
Other type	637 (49.8)	NA	601 (52.0)	NA	41 (33.3)	
Patients with preprocedure VWD panel (n = 730)						
Normal	612 (83.8)	NA	NA	526 (86.6)	86 (69.9)	<.001
Low	104 (14.2)	NA	NA	71 (11.7)	33 (26.8)	
Disease	14 (1.9)	NA	NA	10 (1.6)	4 (3.2)	

Abbreviations: BT, blood type; NA, not applicable; VWD, von Willebrand disease.

**Table 2.** Bleeding Rates by Testing Category

Characteristic	Patient category									
	All		No BT and no VWD panel		BT and no VWD panel		No BT and VWD panel		BT and VWD panel	
	No./total No. (%)	*P* value	No./total No. (%)	*P* value	No./total No. (%)	*P* value	No./total No. (%)	*P* value	No./total No. (%)	*P* value
Total with bleeding	578/14 951 (3.9)	NA	362/13 065 (2.8)	NA	172/1156 (14.9)^a^	NA	28/607 (4.6)	NA	16/123 (13.0)^a^	NA
Age, y										
≤12	423/13 091 (3.2)	<.001	265/11 577 (2.3)	<.001	130/906 (14.3)	.37	16/518 (3.1)	<.001	12/90 (13.0)	.67
>12	155/1860 (8.3)		97/1488 (6.5)		42/205 (16.8)		12/89 (13.5)		4/33 (12.1)	
Sex										
Male	326/7994 (4.1)	.16	215/6994 (3.1)	.02	88/602 (14.6)	.80	11/329 (3.3)	.12	12/69 (17.4)	.06
Female	252/6956 (3.6)		147/6070 (2.4)		84/554 (15.2)		17/278 (6.1)		4/54 (7.4)	
VWD status										
Normal	35/612 (5.7)	.24	NA	NA	NA	NA	22/526 (4.2)	.22	13/86 (15.1)	.20
Low	7/104 (6.7)		NA		NA		5/71 (7.0)		2/33 (6.0)	
Disease	2/14 (14.3)		NA		NA		1/10 (10.0)		1/4 (25.0)	
BT										
O	94/637 (14.6)	>.99	NA	NA	85/555 (15.3)	.74	NA	NA	9/82 (11.0)	.14
Other	94/642 (14.8)		NA		87/601 (14.5)		NA		7/41 (17.0)	

Abbreviations: BT, blood type; NA, not applicable; VWD; von Willebrand disease.

^a^ Includes patients with BT prior to surgery (bleeding rate, 4.8%) and those with BT the day of or within 1 month of surgery (bleeding rate, 45.3%) (eTable in the Supplement).

In all, 13 065 patients had neither BT nor preprocedure VWD panel testing performed (Table 1 and Table 2). For those children, the bleeding rate was 2.8% (362 patients). In this cohort, being older than 12 years (97 of 1488 [6.5%] vs 265 of 11 577 [2.3%]; *P* < .001) and being male (215 of 6994 [3.1%] vs 147 of 6070 [2.4%]; *P* = .02) were also associated with a higher bleeding rates (Table 2).

A total of 1279 children (8.6%) had a recorded BT in our electronic medical record system. The overall bleeding rate was 14.7% (188 of 1279) in all children with a known BT; 14.8% (94 of 637) of children with BT O vs 14.6% (94 of 643) of children with non-O BT experienced bleeding (*P* > .99). These bleeding rates were significantly higher than those reported for children with unknown BT. Given such an elevated bleeding rate in this cohort, the timing of BT identification was further investigated: specifically, those with BT prior to surgery (927 children [73.3%]) exhibited a 4.8% bleeding rate, while those with BT performed the day of or within 1 month after surgery (316 children [24.7%]) had a 45.3% bleeding rate (eTable in the Supplement). In the 927 children with a BT identified prior to surgery, there was no significant difference in bleeding rates in patients with BT O and non-O BT (26 of 461 [5.6%] and 18 of 466 [3.9%], respectively; *P* = .22)

A total of 607 patients who had a VWD panel prior to surgery could be characterized into a final VWD disease category but had no BT in our electronic medical record system. The bleeding rate for these individuals was 4.6% (28 patients), which was significantly greater than the bleeding rate of 2.8% (362 patients) in the untested cohort (*P* = .02). Bleeding rates were also associated with VWD status, with those with normal VWD panels having the lowest bleeding rate (4.2% [22 of 526]) and those with disease having the highest (10.0% [1 of 10]). Those with low VWF had an intermediate bleeding rate of 7.0% (5 of 71 patients) (Table 2). These differences, however, were not statistically significant. Sex was not a risk factor for hemorrhage, but children older than 12 years again had an increased bleeding rate compared with children aged 12 years or younger (12 of 89 [13.5%] vs 16 of 518 [3.1%]; *P* < .001).

A total of 123 children had a BT identified in addition to a VWD panel performed prior to surgery. In this cohort, the bleeding rate was 13.0%. While the BT O group had lower mean (SD) levels of VWF:Ag (86.9 [42.4] IU/dL vs 118.0 [53.8 IU/dL in the non-O group; *P* = .002) and VWF:RCo (72.2 [44.3] IU/dL and 112.6 [68.0] IU/dL in the non-O group; *P* = .001) (Table 3), there was no significant difference in postoperative bleeding incidence between the O and non-O BT groups (Table 2).

**Table 3.** von Willebrand Factor and Coagulation Factor VIII Values by Blood Type

Measure	Patients with blood type O				Patients with non-O blood type			
	All (n = 82)	With bleeding (n = 9)	Without bleeding (n = 73)	*P* value	All (n = 41)	With bleeding (n = 7)	Without bleeding (n = 34)	*P* value
VWF:Ag, mean (SD), IU/dL	86.9 (42.4)	73.3 (20.7)	88.6 (44.2)	.09	118.0 (53.8)	124.6 (62.4)	116.6 (52.8)	.76
VWF:RCo, mean (SD), IU/dL	72.2 (44.3)	62.6 (28.6)	73.4 (45.8)	.15	112.6 (68.0)	111.9 (58.7)	112.8 (70.5)	.97
Factor VIII, mean (SD), IU/dL	100.3 (42.4)	98.6 (29.3)	100.5 (42.8)	.86	116.9 (42.8)	108.3 (40.1)	118.6 (43.7)	.56
VWF category, No. (%)								
Normal	50 (60.9)	6 (66.7)	44 (60.3)	.32	36 (87.8)	7 (100)	29 (85.3)	>.99
Low	29 (35.4)	2 (22.2)	27 (37.0)		4 (10.0)	0	4 (11.8)	
Disease	3 (3.7)	1 (11.1)	2 (2.7)		1 (2.4)	0	1 (2.9)	

Abbreviations: VWF:Ag, von Willebrand factor antigen; VWF:RCo, von Willebrand factor ristocetin-cofactor.

Of the 123 children with a BT and VWD panel prior to surgery, only 37 were found to have low VWF or VWD values. Three of the 37 (8.0%) experienced bleeding after their procedure. Of the 33 children with low VWF, 19 were treated with either desmopressin (DDAVP or 1-deamino-8-D-arginine vasopressin), Humate-P, and/or an antifibrinolytic agent, while 14 were not treated. Ten of the 14 who were not treated had a repeated VWD panel with results that fell within the normal range. The other 4 were not treated secondary to clinician preference. In total, 2 children (5.4%) with low VWF had bleeding. Both patients had a family history of bleeding but no personal history. Both underwent tonsillectomy secondary to sleep disturbance.

A logistic regression estimated the independent associations of age, sex, VWD status, and BT with hemorrhage following surgery. Each factor was statistically significant. Being older than 12 years was associated with greater likelihood of hemorrhage than being 12 years or younger (odds ratio [OR], 2.2 [95% CI, 1.8-2.7]; *P* < .001). Being male was also associated with greater odds of hemorrhage (OR, 1.3 [95% CI, 1.1-1.5]; *P* = .01). All 3 VWD categories were associated with increased likelihood of hemorrhage compared with unknown VWD status (low VWF vs unknown: OR, 3.2 [95% CI, 1.9-5.5]; *P* < .001; normal VWF vs unknown: OR, 4.4 [95% CI, 3.5-5.6]; *P* < .001; and disease vs unknown: OR, 3.9 [95% CI, 0.8-18.3]; *P* < .001). The 3 known VWD status groups did not significantly differ from each other. Similarly, known BT was associated with greater odds of hemorrhage compared with unknown BT (BT O vs unknown: OR, 4.5 [95% CI, 3.5-5.8]; *P* < .001; BT non-O vs unknown: OR, 4.9 [95% CI, 3.8-6.3]; *P* < .001). The 2 BT groups were not significantly different from each other (BT non-O vs BT O: OR, 1.09 [95% CI, 0.8-1.5]; *P* = .60).

## Discussion

In this study, we analyzed factors associated with postoperative bleeding in a large cohort of pediatric patients undergoing tonsillectomy with or without adenoidectomy. With more than 1000 of these procedures being performed at our institution annually, the rate of postoperative hemorrhage was 3.9% for all patients. This is comparable to bleeding rates following tonsillectomy in children with both normal and abnormal coagulation in other published studies^18,19^ but higher than rates seen in a large cohort studied from 2001 to 2010.^2^

Our institutional practice involves the use of a preoperative bleeding questionnaire followed by limited screening testing, which does not include BT, for those with a personal or family history of bleeding. This is consistent with standard recommendations from the American Society of Hematology and American Society of Pediatric Hematology and Oncology, both of whom advocate against routine preoperative testing in children without a personal or family history of bleeding.^20^ Their recommendation is consistent with the reliability of coagulation testing to identify a clinically significant abnormality^21,22,23^ and the unfavorable cost-effectiveness of both targeted and routine screening.^24^

The baseline bleeding rate (in patients with no known risk factors for bleeding or prior pathologies that may affect bleeding complications) in our study was 2.8%. Testing for BT appeared to identify a group of patients (8.6% of the total) with higher bleeding risk, since the bleeding rate in this group of 1279 patients was significantly higher (14.7%). However, the bleeding rate is being largely driven by BT performed the day of or within 1 month after surgery, as the bleeding rate for that group was 45.3%, while those with a BT identified prior to surgery had a bleeding rate of 4.8% (*P* < .001) (eTable in the Supplement). Those with a BT the day of surgery or within 1 month of surgery had BT testing most often owing to postprocedure bleeding.

Testing for BT is not part of routine preprocedure pediatric care and was not performed in 91% of our patients. Of the 1279 children with a BT, there was no difference in the incidence of postoperative hemorrhage in those patients with BT O and non-O BT, suggesting that BT O by itself is not a risk factor for mucosal bleeding after tonsillectomy.

Individuals with BT O have lower VWD panel values than those with non-O BT,^25^ which has fueled research questioning the association between BT O and periprocedural bleeding. Several studies^10,11,12,26^ have identified a bleeding risk in patients with BT O. Two studies^11,26^ have demonstrated an overrepresentation of individuals with BT O in patients presenting with epistaxis. Similarly, Leonard et al,^12^ in a cohort of 206 patients who experienced hemorrhage after tonsillectomy, found a significantly higher proportion of individuals with BT O in their cohort (63%) vs the general population (55%). We therefore hypothesized that bleeding would be increased in our BT O cohort and VWF values would be lower.

While VWF values were lower in our BT O cohort, bleeding rates in our BT O and BT non-O groups were equal. One possible explanation for this is that our cohort had relatively high mean VWF:Ag and VWF:RCo values. However, bleeding secondary to low VWF values is likely to occur only at values below 50%. Means in our cohort were greater than 60% for both VWF:Ag and VWF:RCo. Some of the patients in our cohort with the lowest VWF values were treated preoperatively, which may have influenced our results. However, even when the 21 preoperatively treated patients were not included in our analysis, the bleeding rate for both cohorts remained approximately 15%. Furthermore, VWF values may not be predictive of hemorrhage after tonsillectomy. A recent study^27^ of approximately 1400 children aged 7 to 17 years who underwent tonsillectomy and a postinduction VWD panel found no correlation between low VWF values (<50 IU/dL) and bleeding after tonsillectomy. Analogously, when we subdivided our cohort of 607 patients who had a baseline VWD panel prior to their procedure into normal, low, and disease, bleeding rates were 4.2%, 7.0%, and 10.0%, respectively, but the difference was not statistically significant. However, the number of patients in the low VWF and disease categories, especially among those who experienced bleeding, was very small and may have influenced our ability to capture any differences. Gill et al^27^ also noted the transient nature of low VWF values, especially in children. Interestingly, we found that nearly one-third of patients who had a known BT and were categorized as low VWF in our study had a subsequent normal VWD panel result prior to their procedure, did not receive treatment based on clinician or family preference, and did not have bleeding postoperatively.

Being older than 12 years and being male were also found to be associated with bleeding rates after tonsillectomy. Independent of those findings, we did not find a difference between the bleeding rates of BT O and non-O BT.

Our study findings suggest that BT should not be added to limited preoperative testing of pediatric patients prior to tonsillectomy with or without adenoidectomy. These data convincingly support other studies^14,15,16,17^ that have shown no risk of bleeding associated with BT O.

### Limitations

A key limitation of our study was its retrospective design. While we had a large cohort of patients who underwent tonsillectomy with or without adenoidectomy, many did not have BT or VWF values assessed prior to surgery, as this is not the standard of care. In addition, of the cohort of patients with BT and VWF values, many did not have VWF values less than 50%. Future prospective studies should make an effort to enroll patients with lower VWF values in order to assess whether BT O and low VWF values together pose a risk for postoperative bleeding.

## Conclusions

This large, retrospective pediatric cohort study assessed the association between BT and bleeding after tonsillectomy with or without adenoidectomy. In this study, we found no difference in bleeding rates for those with BT O compared with those with non-O BT. We found no significant difference in bleeding rates associated with VWD disease severity or differences in VWF values in patients who experienced bleeding compared with those who did not. Unfortunately, our cohort of children with both a known BT and preprocedure VWD panel contained only 123 children, most of whom had normal values. In addition, the most patients with low VWF and VWD were treated preoperatively. We conclude that while BT O was not associated with hemorrhage following tonsillectomy in this study, more studies are needed to determine the true value of the preoperative VWD panel assessment for patients undergoing this procedure, particularly in those with low VWF.

## Notes

Supplement. **eTable.** Bleeding Rates According to Timing of Blood Group Determination
