# Supplementary material for: Association of Blood Type With Postsurgical Mucosal Bleeding in Pediatric Patients Undergoing Tonsillectomy With or Without Adenoidectomy
Source: JAMA Netw Open. 2020 Mar 31;3(3):e201804. doi: 10.1001/jamanetworkopen.2020.1804 (PMC7109594; doi:10.1001/jamanetworkopen.2020.1804)
Supplement: Supplement. — eTable. Bleeding Rates According to Timing of Blood Group Determination [file jamanetwopen-3-e201804-s001.pdf]

Supplementary Online Content

Archer NM, Forbes PW, Dargie J, et al. Association of blood type with postsurgical mucosal bleeding in pediatric patients undergoing tonsillectomy with or without adenoidectomy. *JAMA Netw Open*. 2020;3(3):e201804.
doi:10.1001/jamanetworkopen.2020.1804

eTable. Bleeding Rates According to Timing of Blood Group Determination

This supplementary material has been provided by the authors to give readers additional information about their work.

© 2020 Archer NM et al. *JAMA Network Open.*

eTable: Bleeding rates according to timing of blood group determination

**Category**	**N**	**Bleeding rate**	**% Bleeding rate by age**			**% Bleeding rate by sex**			**% Bleeding rate by VWD status***				**% Bleeding rate by BT**		
		All	≤12	>12	*P* value	Male	Female	*P* value	normal	low	disease	*P* value	BT O	BT non-O	*P* value
BT known, (%) No.	1279	14.7 (188)	No. 996<br>14.3 (142)	No. 283<br>16.3 (46)	.39	No. 671<br>14.9 (100)	No. 608<br>14.5 (88)	.87	No. 86<br>15 (13)	No. 33<br>6 (2)	No. 4<br>25 (1)	.20	No. 637<br>14.8 (94)	No. 642<br>14.6 (94)	1.0
BT prior to procedure, (%) No.	927	4.8 (44)	No. 735<br>4.5 (33)	No. 192<br>5.7 (11)	.45	No. 499<br>4.8 (24)	No. 428<br>4.7 (20)	1.0	No. 81<br>7 (6)	No. 31<br>0 (0)	No. 4<br>25 (1)	.07	No. 461<br>5.6 (26)	No. 466<br>3.9 (18)	.22
BT day of or within 1 month after surgery, (%) No.	316	45.3 (143)	No. 232<br>46.4 (108)	No. 84<br>42 (35)	.52	No. 154<br>48.7 (75)	No. 162<br>42 (68)	.26	No. 49<br>80 (39)	No. 7<br>100 (7)	No. 0<br>0 (0)	NA	No. 159<br>42.8 (68)	No. 157<br>47.8 (75)	.43
BT more than 30 days after surgery, (%) No.	36	2.8 (1)	No. 29<br>3 (1)	No. 7<br>0 (0)	1.0	No. 18<br>6 (1)	No. 18<br>0 (0)	1.0	No. 3<br>0 (0)	No. 3<br>33 (1)	No. 0<br>0 (0)	NA	No. 17<br>0 (0)	No. 19<br>5 (1)	1.0

*Patients may be included in multiple categories depending on number of times BT assessed

VWD - von Willebrand disease
BT - blood type
No. - number

© 2020 Archer NM et al. *JAMA Network Open.*
